# Directional Ultrasound Source for Solid Materials Inspection: Diffraction Management in a Metallic Phononic Crystal

**DOI:** 10.3390/s20216148

**Published:** 2020-10-29

**Authors:** Hossam Selim, Rubén Picó, Jose Trull, Miguel Delgado Prieto, Crina Cojocaru

**Affiliations:** 1 Physics Department, Universitat Politècnica de Catalunya, Rambla Sant Nebridi 22, 08222 Terrassa, Spain; hossam.eldin.mohamed.selim@upc.edu (H.S.); jose.francisco.trull@upc.edu (J.T.); crina.maria.cojocaru@upc.edu (C.C.); 2 Instituto de Investigación Para la Gestión Integrada de Zonas Costeras, Universitat Politècnica de València, Paranimf 1, Grao de Gandia, 46730 València, Spain; 3 Electronic Engineering Department, Universitat Politècnica de Catalunya, Rambla Sant Nebridi 22, 08222 Terrassa, Spain; miguel.delgado@upc.edu

**Keywords:** phononic crystals, self-collimation, ultrasonic lens, acoustic lens, ultrasonic wave diffraction, NDT

## Abstract

In this work, we numerically investigate the diffraction management of longitudinal elastic waves propagating in a two-dimensional metallic phononic crystal. We demonstrate that this structure acts as an “ultrasonic lens”, providing self-collimation or focusing effect at a certain distance from the crystal output. We implement this directional propagation in the design of a coupling device capable to control the directivity or focusing of ultrasonic waves propagation inside a target object. These effects are robust over a broad frequency band and are preserved in the propagation through a coupling gel between the “ultrasonic lens” and the solid target. These results may find interesting industrial and medical applications, where the localization of the ultrasonic waves may be required at certain positions embedded in the object under study. An application example for non-destructive testing with improved results, after using the ultrasonic lens, is discussed as a proof of concept for the novelty and applicability of our numerical simulation study.

## 1. Introduction

The ability to manage the diffraction of ultrasound waves has been a subject of study for decades in many applications, such as radar, sonar, theaters, echography, etc. [1,2]. The term “acoustic lens” is commonly used for materials (usually artificially designed) that are able to change the diffraction properties of the propagating acoustic waves [2,3,4]. In the last two decades, phononic crystals (PC), which are periodic arrangements of two different materials with different acoustic parameters and a periodicity of the order of the wavelength, have proven to be a convenient solution for the management of sound waves diffraction [5,6]. The dispersion relation of the propagation modes within the PC is modified by the periodic modulation of the medium. As a consequence, wave propagation in PCs can lead to interesting physical phenomena like wave convergence, divergence, or focusing, depending on the physical parameters of the medium, geometrical configuration, and the ultrasound wavelength [5,6,7,8]. Of particular interest, the self-collimation effect is produced when ultrasound waves can propagate a certain distance behind the PC without diffraction broadening [7], keeping a constant diameter and increasing the directivity [8]. By means of a PC, one can design an ultrasonic lens (UL) that can control the propagation in different materials for different applications.

Diffraction control of acoustic waves when they propagate in a PC has been proven in different theoretical and experimental studies for propagation in linear [9] and nonlinear [10] fluids and in solids. Notwithstanding, the management of diffraction in solid media has been less studied. To our knowledge, no studies, or maybe very limited ones, have been conducted on the propagation of ultrasound in metals after leaving PCs. In solids, we have longitudinal and shear wave propagation in comparison with fluids where only longitudinal waves propagate. For example, a multifunctional solid (steel)/solid (epoxy) PC was shown to perform a variety of spectral, wave vector, and phase-space functions, including acoustic wave collimation, a defect-less wave guide, and a directional source for elastic waves [11]. The propagation of sound beams beyond PCs has been studied using numerical methods showing focusing characteristics [12]. Serkan Alagoz experimentally studied intensity maps of far-field focusing using a two-dimensional (2D), triangular lattice sonic crystal, and intensity maps of near-field focusing using a 2D square lattice PC for various scattering materials [9]. Morvan et al. used a 2D PC-based solid/solid composite to demonstrate experimentally and theoretically the spatial filtering of a monochromatic, non-directional wave source and its emission in a surrounding water medium as an ultra-directional beam with narrow angular distribution [13]. C. Charles et al., investigated the propagation of elastic waves in 2D PC by using plane wave expansion method to calculate the dispersion relations of guided elastic waves in these periodic media, including 2D phononic plates and thin layered periodic arrangements [14]. Ralf Lucklum studied the ability to magnify the interaction of acoustic waves with matter through specific geometries that are capable of concentrating acoustic waves in a confined volume forms the basis of a novel type of biosensor [15]. Jimenez et al. reported the nonlinear focusing of ultrasonic waves by an axisymmetric diffraction grating immersed in water. They showed numerically and experimentally that the focal gain of the system increases extraordinarily in the nonlinear regime [16]. Ye et al. studied the acoustic wave propagation in liquid media with air bubbles. The authors have derived a set of coupled equations describing rigorously the multiple scattering of waves in such media [17]. Sharma et al. designed a directivity-based barrier for the local control of low-frequency noise using a point mass attachment on the barrier surface [18]. They showed analytical equations for the vibration response and sound transmission through the unloaded and mass-loaded plates. They also presented a concept of local noise control in specific directions of interest using a model problem of a simply-supported plate in a baffle [19]. Local noise control is achieved by altering the directivity of sound radiation of the source using a point mass attachment. Leroy et al. showed that acoustic super absorption can be achieved using a meta-screen based on a periodic arrangement of bubbles embedded in a soft elastic matrix [20].

In this work, we propose a 2D metallic PC device that coupled to an ultrasound transducer is able to manage the diffraction of ultrasonic longitudinal elastic waves. We aim to control and enhance the directivity of ultrasound waves further propagating in a homogeneous solid target placed behind the PC, with a benefit for different applications where directional acoustic wave propagation is needed in a certain position of the sample. We consider a 2D PC, consisting of a square lattice periodic distribution of cylindrical air holes drilled in aluminium. Through detailed numerical simulations, we analyse the dispersion relation and spatial distribution of the wave at the output and behind (in the far field) of the PC. The parameters are carefully chosen to provide specific patterns based on physical diffraction effects like focalization and self-collimation. In the next step, we simulate and evaluate a realistic situation where the broadband frequency ultrasound is emitted by an ultrasound transducer, spatially filtered by the PC, and coupled to the object under inspection. Finally, we discuss a possible application where PC lens can be used for NDT applications to detect cracks in solid materials with an improved response.

**Figure 1:**
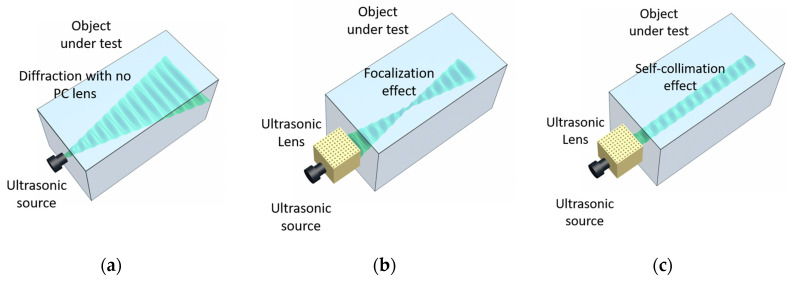
Schematic representation of ultrasound propagation beam into the object under test. Beam diffraction of the ultrasonic source when it is directly coupled to the sample (**a**), coupled to the object under test through acoustic lens focusing waves inside the object (**b**) and coupled through an acoustic lens generating self-collimation of waves inside the object (**c**).

A schematic representation of the proposed PC device, together with the target under study, is shown in Figure 1. When a typical transducer is directly coupled to a solid target the acoustic beam is strongly diffracted, resulting in a fast spatial spreading of the wavefront (Figure 1a). One possible solution to reduce the diffraction is to insert a 2D PC acting as an acoustic lens, capable of modifying and controlling the waves propagation, between the transducer and the object under study. Depending on the properties of the PC and on the transducer’s frequency band, two propagation regimes are possible behind the PC: focusing (Figure 1b) and self-collimation (Figure 1c).

## 2. Phononic Crystal Dispersion Band Analysis

Propagation properties of the acoustic waves in a PC are determined by the acoustic parameters of the medium, geometry, constant and filling factor of the periodic lattice, and by the wavelength. The dispersion relation of a PC is a useful representation of the propagating modes in the reciprocal space [6,21,22]. For certain geometries and propagation directions, frequency band gaps may appear in a specific frequency range where acoustic wave propagation is forbidden inside the crystal. Another useful representation is the equifrequency contour maps, ω(*k_x_*,*k_y_*), (also known as equifrequency surfaces (EFS)), which gives information about the propagation of the acoustic beams at different frequencies in different directions inside the PC. The acoustic waves propagate inside the crystal as a set of plane waves and Bloch modes at different angles. The direction of the energy flow for a given wavevector coincides with the normal to the equifrequency contour, pointing towards the increase in frequency [23]. Accordingly, beams projected into convex EFS (with respect to the centre of the unit cell), reduce their diffraction through propagation inside the crystal. This particular behaviour of waves in periodic media leads to focusing. The more tilted the projected segments of the EFS, the more intense the focusing. When flat segments appear in the EFS of propagating Bloch modes, the self-collimation effect is produced inside the crystal. As the curvature of EFS is related to the diffractive broadening of the beams, then the flattening of these lines implies the vanishing of diffractive broadening, i.e., the beam is collimated [8,24,25]. A concave contour line would result in positive diffraction (divergence) [26,27,28,29]. Consequently, under special circumstances, the propagation of narrow beams is possible over long distances without diffractive broadening inside the PC.

**Figure 2:**
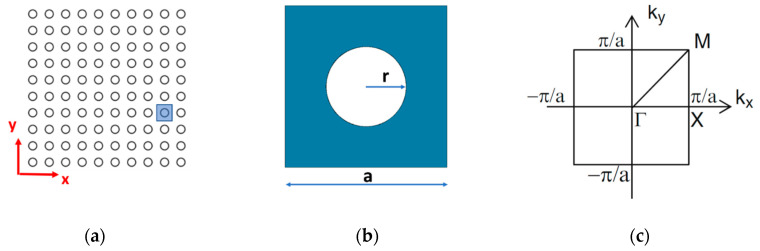
Schematic diagram of PC structure with inset of a unit cell (**a**) the 2D lattice structure (**b**) unit cell representation in real space (x, y), described by the parameters r, a; (**c**) unit cell representation on the reciprocal space (*k_x_* and *k_y_*): Bloch vector in the Γ-X-M directions.

Here, we consider a 2D PC consisting of a periodic distribution of cylindrical holes (air) drilled in an aluminum block, arranged in a square periodic lattice, as schematically shown in Figure 2a. The square unit cell in real space is shown in Figure 2b, where *a* is the lattice constant and *r* is the radius of the air hole. Figure 2c shows the unit cell in the reciprocal space with the Bloch vector representation of the Γ-X-M directions where (*k_x_, k_y_*) are wave vectors in the 2D reciprocal space.

**Figure 3:**
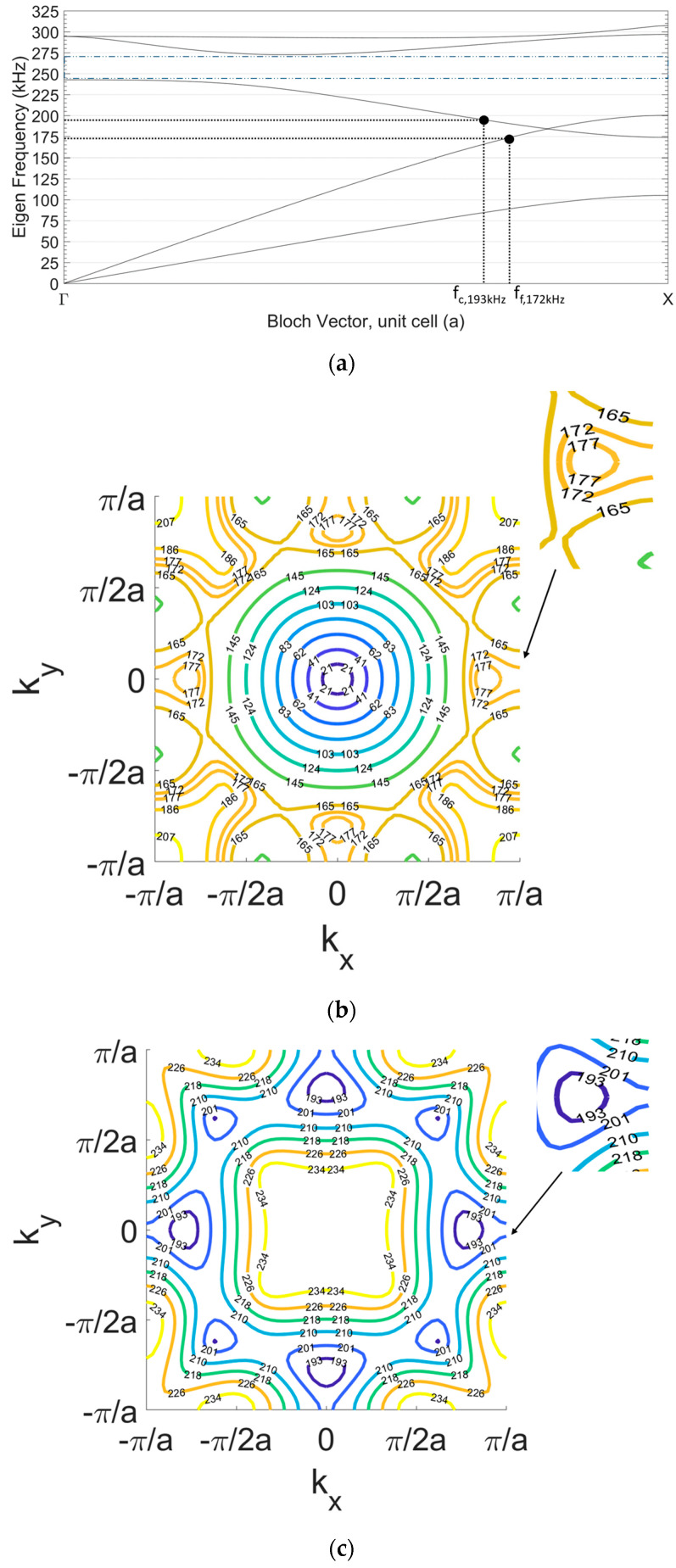
Dispersion characteristics of the 2D periodic PC with square lattice, constant lattice, a = 10 mm and radius of scatterers (holes), r = 2.5 mm. (**a**) Band structure for the Г-X direction. The lower and upper frequency limits of the first bandgap are shown in dashed lines. Equifrequency surfaces (EFS) in the reciprocal space (*k_x_*, *k_y_*) (**b**) for mode 2 and (**c**) mode 3.

In this study we calculated the band structure and equifrequency surfaces of different PCs with a square lattice with different parameters of the lattice constant, hole radius and filling factor. We use COMSOL and MATLAB software to simulate the dispersion relation and to calculate the equifrequency lines. We analysed the results in all the representative propagation directions in the reciprocal space to fit the requirement of an appropriate diffraction pattern for the focusing of waves or collimation in the ultrasonic regime. In our pre-analysis we checked the dispersion curves for radii from 0.5 mm to 4.5 mm. We applied the dispersion curve analysis and iso-frequency calculations as well as frequency response calculations for different hole radii. Afterwards, we chose the hole radius that provided the desired frequency response. Here, we only show the PC with the chosen configuration for focusing and collimation with air holes of radius r = 2.5 mm and lattice constant a = 10 mm, corresponding to a filling factor $ff=\frac{\pi r^{2}}{a^{2}}=0.2$. The band structure of this PC is shown in Figure 3a. For simplicity, only the Bloch vector in the Γ-X direction of the reciprocal space is represented in the horizontal axis, which is the direction of emission. We can see that a bandgap in the Γ-X direction appears between 230 kHz to 270 kHz (both frequency limits are shown with dashed lines). Mode 1 (first curve from bottom) is linear across most of the Γ-X axis, which corresponds to linear propagation with normal diffraction (no dispersion). Mode 2 and mode 3 (second and third curves from bottom) correspond to longitudinal and transverse waves that suffer some dispersion due to the presence of the PC.

Figure 3b,c show the EFS of both modes of interest, mode 2 and mode 3, respectively. Figure 3b shows the EFS for the frequencies of interest where focusing is expected to occur in band 2. This regime is close to the boundary of the band, next to the band-gap, where the dispersion curves are convex and the focusing of the beams behind the crystal is possible [30]. We note that the equifrequency curves present concave segments along the Г-X direction (*k_y_* = 0) in Mode 2 for frequencies above 165 Hz. More specifically, it can be seen that the focusing occurs with a convex shape of EFS and a wider spatial width at frequency f_f_ = 172 kHz. Figure 3c shows the EFS of band 3 where flat segments can be observed around 200 kHz at the Г-X direction. As propagation out of the crystal bends the surfaces towards concavity, it will be shown that the frequency of collimation is found at f_c_ = 193 kHz.

## 3. Non-Diffractive Wave Propagation

**Figure 4:**
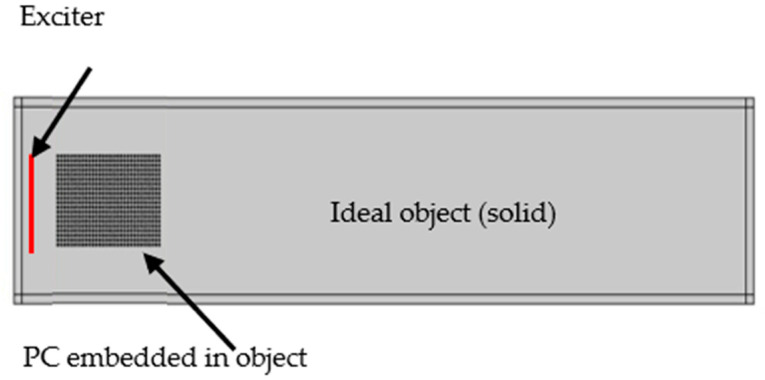
Layout of piston-like source-PC simulation model. Here both the piston and the PC are embedded in the solid target material.

Now, we consider only the wave propagation projected in mode 2 where the curvature of the equifrequency lines is convex (corresponding to the focusing effect), or flat (corresponding to the self-collimation effect). Elastic equations are solved in the solid media by discretizing the medium with simulations based on the finite element method, using COMSOL software with aluminium as the solid media and air as the holes in the PC. We used a perfectly matched layer to avoid any reflection at the boundaries. Due to the very high impedance contrast between aluminium and air, the scatterers are assumed to reflect elastic waves perfectly. Therefore, it is assumed that there is no propagation in the air. We simulate the frequency-dependent propagation of an ultrasonic beam excited by a piston-like source as shown in the layout in Figure 4, with the PC inserted at 5.5 cm spacing from the source. Even if this is not a realistic situation; in this first simulation, both the piston-like source and the PC are considered to be embedded in the same block of aluminium and host medium is assumed to be homogenous. We start with this simple configuration to better illustrate the effect of the PC on the acoustic wave propagation at different frequencies.

The input source was simulated as a prescribed displacement source with an amplitude only in the horizontal direction. The displacement field x is composed by two components x(u,v). We applied calculations for surface plot of the absolute value of the horizontal displacement field abs(u) at different frequencies, the direction of the excitation. In our configuration, the horizontal displacement represents the propagation of longitudinal waves.

The analysis is performed with COMSOL using a frequency domain analysis with a harmonic regime. We used the following parameters for this simulation analysis. The speed of ultrasound in aluminium is 6300 m/s and a speed of sound in air is 343 m/s. We considered the maximum possible frequency for our analysis to be 250 kHz, which corresponds to minimum wavelength λ_Al_ = 25 mm for the solid material and λ_air_ = 1.4 mm for the air holes. We selected a triangular mesh such that the maximum mesh element size is considered to be λ_Al_/8 for the aluminium object. For the PC mesh size, we used a maximum mesh element size λ_air_/8 to eliminate any numerical error resulting from the small hole radius boundaries. We used solid mechanics physics for the solid material assuming it to be isotropic, we used pressure acoustics physics for the air sections assuming it to be linear elastic material, and we used the Acoustic structure interaction multiphysics to control the interaction between solid and air boundaries. In all outer side boundaries, we used perfectly matched layers, while for inner boundaries between solid/air we used the acoustic/structure coupling boundary condition at the interface. At the beginning, we inserted the input source, in the form of a prescribed displacement in the horizontal direction, u = 1 cm, inside the solid domain. For the FEM calculation, we used a stationary resolver. The convergence criteria considered the relative tolerance to be 0.001. Material losses due to friction and temperature were not considered in this analysis. We note that our phononic crystal considers air holes in a metal host where the high impedance contrast between both media prevents any resonance effect. For this analysis, we used the linear elastic solid equations in the PC host and the target metal object and the Helmholtz equation in the air holes and the coupling gel that are all built-in equations in COMSOL software for the mentioned parameters, domains and physics. We did not add any custom equations to the model. More details of these equations are available and can be found in [31,32].

**Figure 5:**
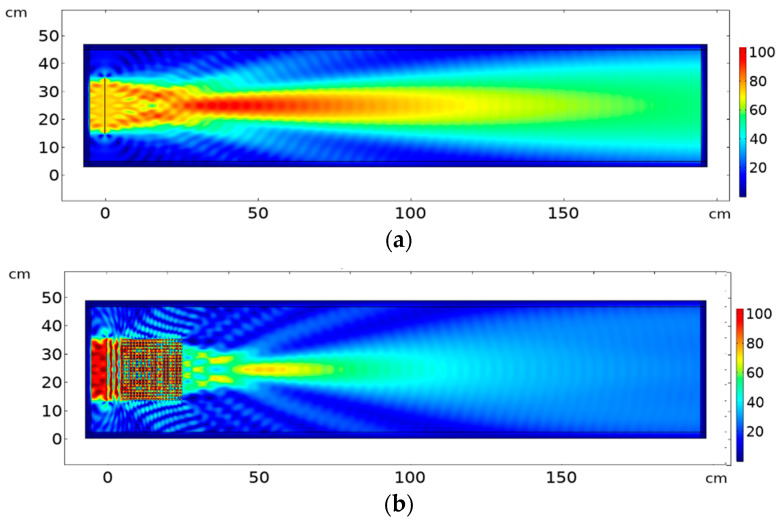

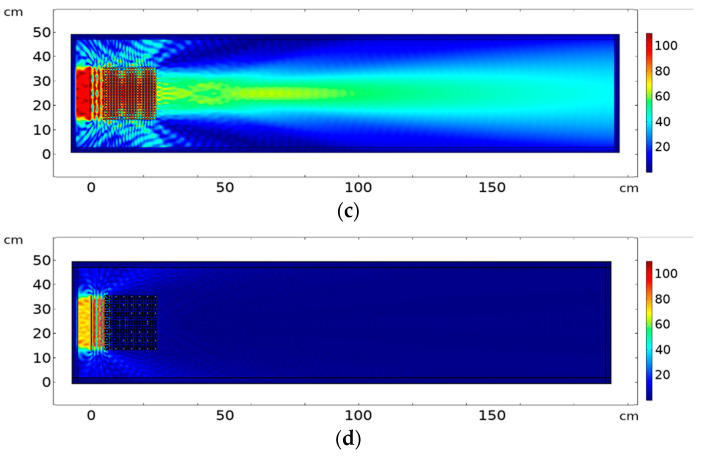
Surface plot of absolute of horizontal output displacement (u) propagation using a PC with 20 × 20 elements, a piston-like line excitation source size 20 cm as input displacement (u), and the spacing between source and the phononic crystal is 5.5 cm. Detection frequency: (**a**) 172 kHz (no crystal) (**b**) 172 kHz with PC (focusing) (**c**) 193 kHz with PC (collimation) (**d**) 250 kHz with PC (bandgap). Color map represents the percentage of the maximum value of horizontal displacement abs(u), i.e.,: intensity at the surface plot is normalized to the maximum intensity in the plot.

Figure 5 shows the different propagation regimes obtained for different frequencies. Figure 5a shows the free propagation of the ultrasound emitted by the source at the frequency of 172 kHz in free propagation from the source to the medium (no periodic modulation is present). From the band structure of the PC shown in Figure 3 one may surmise that the second mode of propagation (longitudinal) is active up to 200 kHz, where the band-gap appears. Figure 5b shows the surface plot intensity at the same 172 kH frequency, when the PC is inserted in front of the source. The focusing occurs with maximum intensity at a distance of 15 cm from the crystal output and a rather constant focalization diameter for a propagation distance of ~25 cm, after which diffraction is similar to propagation in the homogeneous metal. Figure 5c corresponds to a collimation effect obtained at the frequency of 193 kHz that matches with the contour map in Figure 5. Within the bandgap, there is a complete diminishing of the wave amplitude at frequency 250 kHz, as shown in Figure 5d, which matches the dispersion curves in Figure 3a. The colour map in Figure 5 and in all surface plots described next in this chapter represent the percentage of the maximum value of horizontal displacement abs(u), i.e., intensity at surface plot is normalized to the maximum intensity in the plot.

Any slight alteration of the PC filling factor size would result in a slight variation in the operating frequency of focusing or self-collimation. we have applied some alteration to the filling factor in the PC structure by changing the radius of hole inclusions. We found that the same focalization/collimation results take place with a slight variation in the operating frequency, that is a well-known feature of the PC properties. We altered the original hole width from 0.25 mm to 0.24 mm and 0.26 mm respectively. We simulated the surface plot response of the intensity abs(u), the resulting frequencies for focusing and self-collimation have changed for r = 0.24 mm to be 174 kHz, 198 kHz respectively, and for r = 0.26 mm to be 170 kHz, 190 kHz respectively. In case this slight change is encountered due to the fabrication process, it is required to do a calibration sheet for the built device as a commonly known strategy in all fabrication processes in industry. Once PC is fabricated, no change will happen in its own parameters.

**Figure 6:**
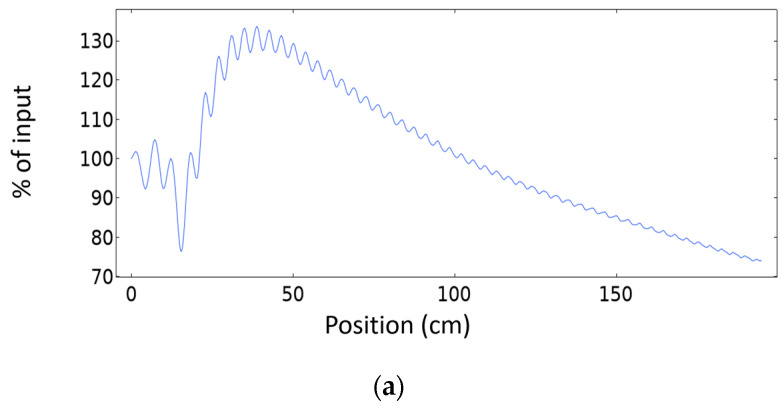

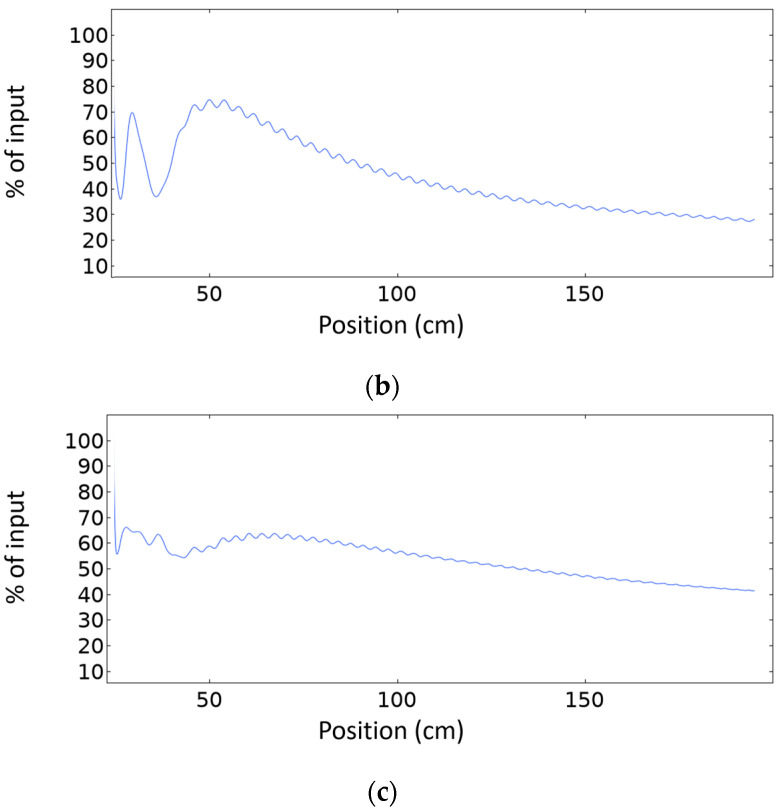
Absolute of horizontal output displacement abs(u) propagation across horizontal line passing by the center of object from the source (position = 0) to the end of the geometry, in case of (**a**) no crystal and at frequency f = 172 kHz (**b**) 20 × 20 PC and at frequency f_f_ = 172 kHz. (**c**) 20 × 20 PC and at frequency at f_c_ = 193 kHz.

In order to better visualize the evolution of the beam diameter across the propagation distance behind the PC in Figure 6 we represent the amplitude, measured across a horizontal line at the center of the crystal passing from left to right. Figure 6a shows the response in case of free propagation at 172 kHz without the PC. The natural oscillation can produce some focalization due to the interference produced by the line source, but it is much broader than the case when a PC is used. Figure 6b shows the horizontal axial profile of the horizontal displacement for the surface geometry at 172 kHz when a PC is used. Once the waves exit the PC the focalization response produced by the PC geometry can be observed. The percentage of maximum intensity at the focal point to source intensity in case of free propagation and using PC are 133% and 75%, respectively. The insertion loss of the PC resulted in the focalization intensity to be 75% of input intensity. The focalization appears at a distance between horizontal line X = ~45 to ~70 cm in both Figure 5b and Figure 6b. In Figure 6c, we observe that at the collimation frequency the horizontal displacement is smooth across the horizontal line passing at the center of the object with a small intensity variation of 15% for a distance of 120 cm inside the target metallic structure. The percentage of collimation intensity to source intensity is ~65% at 193 kHz. This is in comparison with the aforementioned 75% maximum focalization intensity percentage (only at focusing point, and amplitude is decreasing rapidly with distance (Figure 6b) in comparison with collimation).

## 4. Ultrasonic Lens Device

**Figure 7:**
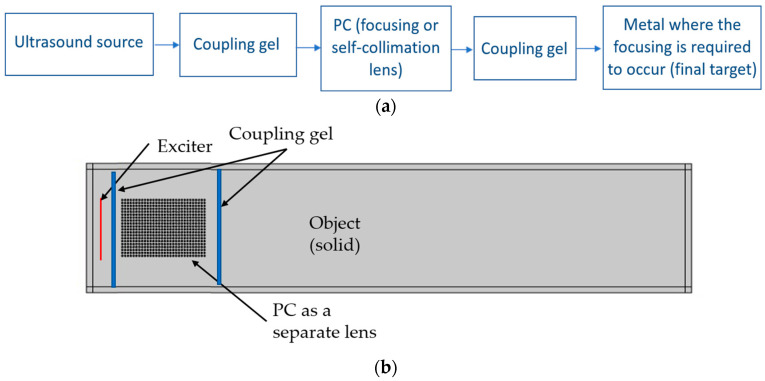
(**a**) Block diagram of the experimental configuration where PC is used as a focusing or a collimation lens that is inserted halfway between ultrasonic transducer and object under test. An acoustic coupling gel is inserted at the interface between source, PC lens and solid object, (**b**) layout of simulation model for the case of using PC lens attached to metallic structure with coupling.

As shown in Figure 4, the aforementioned analysis was performed considering that the PC is built in the same aluminum block and it is part of the same domain of propagation behind the PC. However, this does not match a real experimental configurations, where the excitation is done through an ultrasonic source (e.g., transducer) coupled to the PC (which may be considered as an acoustic beam-shaping lens for the purpose of focusing or collimation effect on the target object) using a coupling material (fluid gel with acoustic impedance matching with the material of propagation). The PC itself should be coupled to the main solid material where we need to investigate the collimation or focusing effects. As it is usual in non-destructive testing (NDT) applications, the coupling material has the appropriate acoustic impedance to enhance the transmission from the transducer to the solid material where ultrasonic propagation waves take place. In real experiments, the coupling has an acoustic impedance that should be very close to that of the material of interest to reduce coupling losses. Figure 7a shows the block diagram of a possible experimental configuration while Figure 7b shows the schematic layout for the COMSOL simulation model. The model uses acoustic/structure interaction physics that takes into account both propagation in the gel (fluid material) and the PC, and the solid object (solid material). As the source is in a fluid, the input magnitude is a reference acoustic pressure source in a fluid (gel with acoustic impedance 75% of that of the solid material) and not an acoustic displacement as in previous simulations. Along propagation, this wave pressure is transmitted to the solid as a wave displacement field at the fluid-solid interface where appropriate boundary conditions are imposed. The gel coupling thickness before and after crystal is 2 mm. It should be noted that the impedance of the gel might be affected by stress especially because of the high acoustic energy focusing as this could change the coupling losses. Hence, in an actual experimental configuration, the calibration of the PC response should be considered with experimental measurements to highlight its effect [33,34].

For this analysis, the incident input source was placed at the left side and coupled to the gel. The incident field was applied in the gel fluid domain and was controlled by the pressure acoustics physics. It was assumed by a plane wave radiated pressure field in the horizontal direction P = 1000 Pa. The gel material was simulated using custom material parameters with acoustic impedance = 75% of the aluminum material acoustic impedance. A triangular mesh size for the coupling gel was λ_air_/8. Acoustic/structure coupling boundary condition was used at the interface between solid material and coupling gel. All other physics parameters are similar to those mentioned in Section 3.

**Figure 8:**
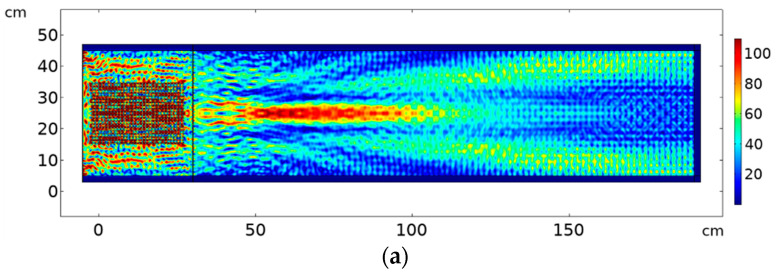

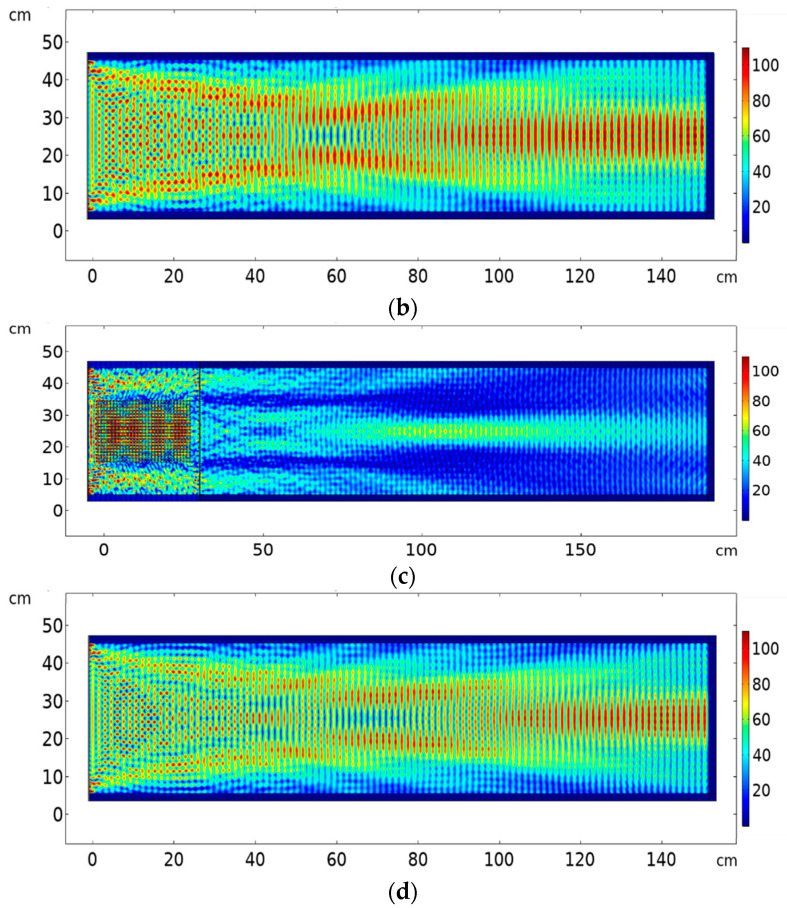
Using 30 × 20 element PC lens with coupling interface between source, lens and object for (**a**) focusing with PC at 172 kHz; (**b**) propagation at 172 kHz without PC; (**c**) collimation with PC at 193 kHz; (**d**) propagation at 193 kHz without PC.

Figure 8 shows the results of propagation through a 30 × 20 element PC used as a focusing/collimation lens. The focusing/collimation effect of the PC is frequency-dependent, as pointed out earlier. The width of the input source of excitation and the PC lens is the same as the object under test (i.e., they cover all the object surface area). The wave field in the solid is blurred by a pattern of fringes that is due to the multiple reflections of waves in the coupling layers. Figure 8a shows a clear focalization effect obtained at 172 kHz, in comparison with Figure 8b where the same frequency propagates in aluminium in the absence of the PC. On the other hand, the collimation effect is observed at the frequency of 193 kHz with effect of PC (Figure 8c), in comparison with the same situation with no PC (Figure 8d).

**Figure 9:**
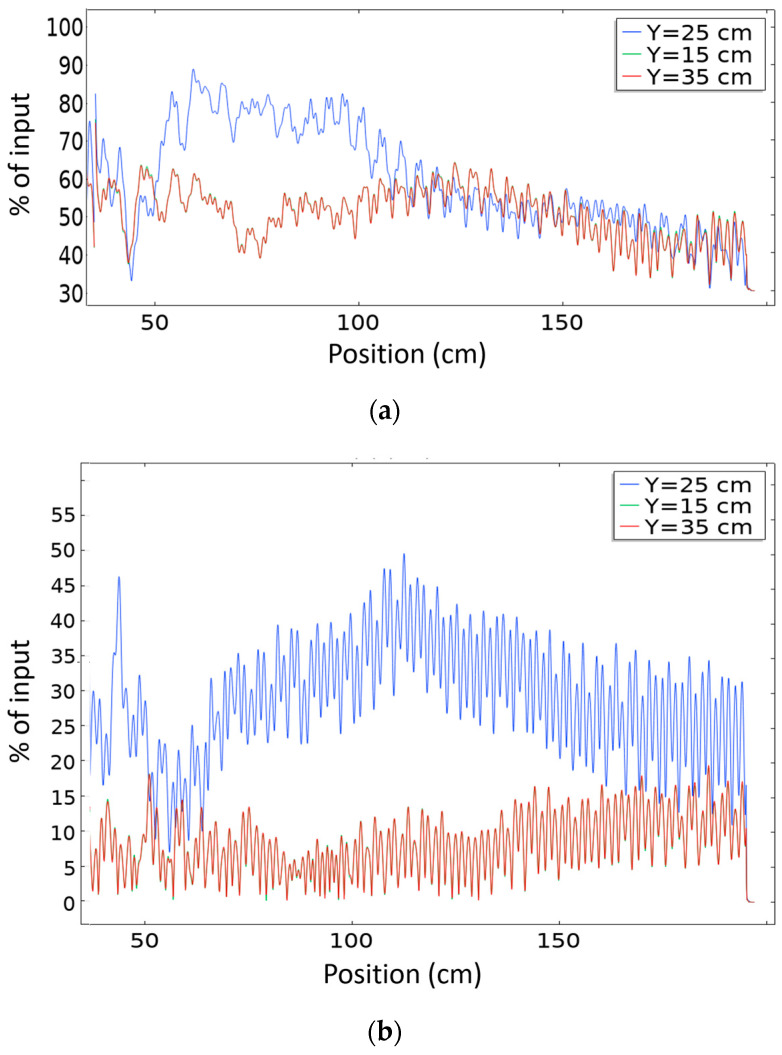
Absolute of horizontal output displacement (u) propagation across horizontal line at Y = 25 cm passing by center of PC lens of 30 × 20 elements to the end of the geometry, in case of (**a**) frequency 172 kHz (focusing regime), (**b**) frequency 193 kHz (collimation regime).

Figure 9 shows the intensity across a horizontal line passing through the centre of the PC (Y = 25 cm) for the same surface plot representation in Figure 8. Figure 9a shows the response in the focusing regime (172 kHz); it is clear that the intensity drops with distance from the focal point. When the horizontal line shifts up or down (Y = 15 cm or Y = 35 cm, red curve), the intensity is low at short distances, because there is only focusing at the centre of the object (Y = 25 cm, blue curve), but with larger distances the intensity slightly increases due to diffraction effects.

In Figure 9b, the response at frequency 193 kHz (collimation regime), the intensity is almost uniform after the collimation is achieved and the decay happens slowly. In this case, when the horizontal line shifts up or down (Y = 15 cm or Y = 35 cm, red curve), the intensity is very low across the whole object size because all the intensity is concentrated in the centre (Y = 25 cm, blue curve) due to collimation effects.

The intensity ratio between focalization and source intensity at 172 kHz is 90% (Figure 8a and Figure 9a) (source considered just at the entrance to the PC lens after coupling, while the ratio between collimation intensity and source intensity at 193 kHz is 50% (Figure 8c and Figure 9b). Thus, the PC lens effectively focuses or self-collimates the beam with limited insertion loss. Comparing these results with the case of no crystal, we find that for same source intensity, the maximum diffraction intensity abs(u) compared to input intensity at the input to the solid object directly after the coupling gel is 142% at 172 kHz (Figure 8b) and 128% at 193 kHz (Figure 8d).

We note that after using the coupling, wave propagation inside the object has been distorted and diffraction increased significantly compared to the case when no crystal was used (Figure 8b,d). However, when we used the crystal, diffraction was controlled to a great extent in both focalization and collimation regimes (Figure 8a,c). This is a great enhancement of the wave propagation pattern inside the object thanks to the PC lens.

**Figure 10:**
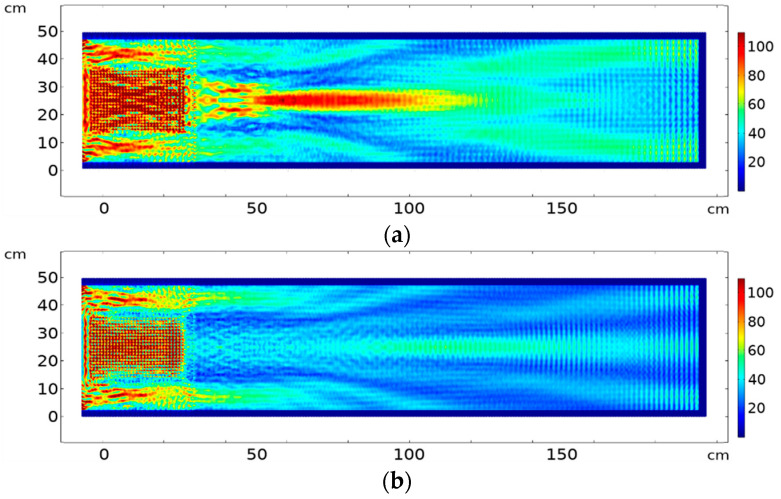
Using 30 × 20 element PC lens with coupling interface between source, lens and object for: (**a**) focusing at frequency bandwidth of 10 kHz with cut-off frequencies 167 to 177 kHz (**b**) collimation at frequency bandwidth of 10 kHz with cut-off frequencies 190 to 200 kHz.

Previous results were simulated at specific single frequencies. In real experiments, ultrasonic transducers that are sensitive in a frequency bandwidth rather than single frequencies are usually used. The question if the focusing and the collimation effect would be preserved when the whole frequency bandwidth is analyzed arises. Figure 10 shows the results for focusing and collimation considering a broader band frequency integration. The results for focusing at frequency bandwidth of 10 kHz, with cut-off frequencies 167 to 177 kHz is shown in Figure 10a. The focusing effect is further enhanced and much stronger compared to the single frequency representation in Figure 8a since the whole bandwidth lies in the focusing zone. Figure 10b shows the results for collimation regime for frequency integration at 10 kHz bandwidth with cut-off frequencies 190–200 kHz. The frequency integration representation is attenuated in comparison with the single frequency surface plot in Figure 8c, because collimation occurs for a very narrow frequency range.

## 5. PC Lens for Non-Destructive Testing Application

As mentioned before, the main goal of this research is the design of an ultrasound source able to reduce the diffraction through collimation or focusing effect obtained in a PC lens. This control of the diffraction can be useful in many applications. As an example, we mention here an NDT applied to the localization of a defect embedded in a metallic sample where source and detector are both mandatory components in the experimental configuration. In this technique, since ultrasound waves usually diffracts in a short propagation distance, the boundary reflections interfere with the waves targeted at the defect location within the specimen. Focusing or self-collimating the incident wave from the source can help to avoid these boundary reflections and the detection of the scattered waves from the defect either in transmission or reflection modes can be done using a cleaner signal with less unwanted perturbations.

**Figure 11:**
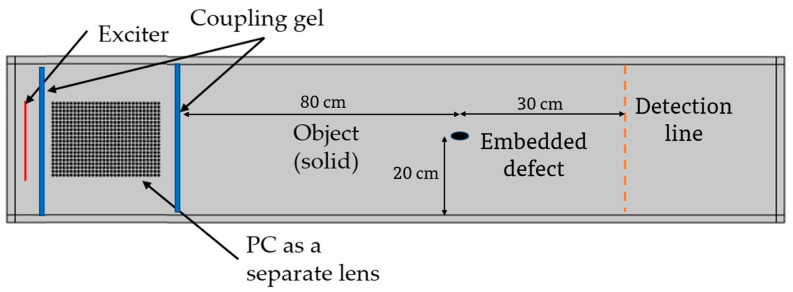
Schematic representation of the material under test attached to the PC lens with the addition of an elliptical air-filled crack at the center of the target material.

We considered the ultrasound source coupled to the PC lens shown and analysed in Figure 7, and we include a defect in the solid target. Figure 11 shows a schematic representation of the defect embedded in the target material. We simulate the ultrasound propagation and we detect the transmitted ultrasound waves at the opposite side of the target (transmission NDT mode).This topology is used in many NDT analyses in the literature as well as beam forming for NDT applications [35,36,37,38,39,40].We compare the results obtained in the case of a healthy sample (shown in Figure 8) with those corresponding to a sample with an elliptical crack (elliptical trigonometric orthogonal axes’ parameters a, b = 1, 2 cm respectively) and simulated as an air filled crack at a horizontal distance of 80 cm from the left border of the aluminium target specimen and vertically positioned at 20 cm in the middle of the specimen with and without using the ultrasound lens formed by the PC.

**Figure 12:**
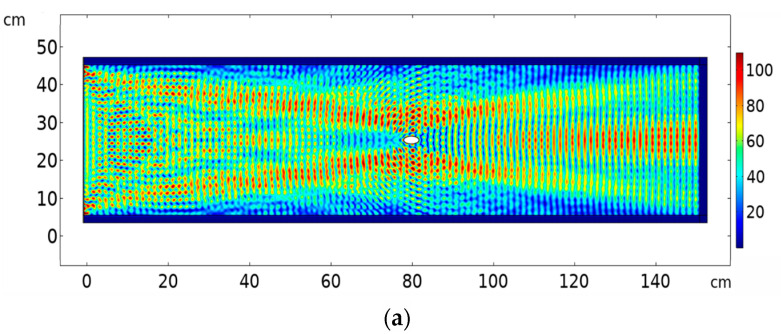

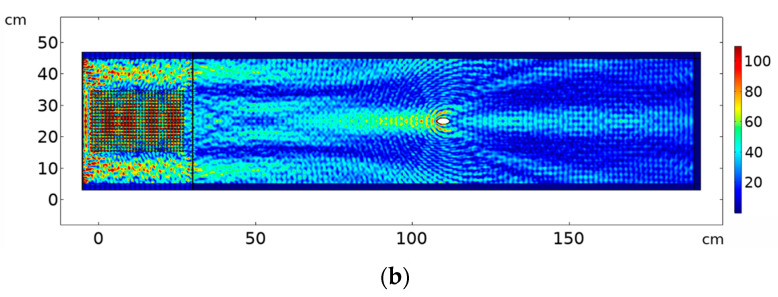
(**a**) surface plot of abs(u) using 30 × 20 element PC lens with coupling interface between source, lens and object for propagation at 193 kHz (**a**) without PC (**b**) with PC.

**Figure 13:**
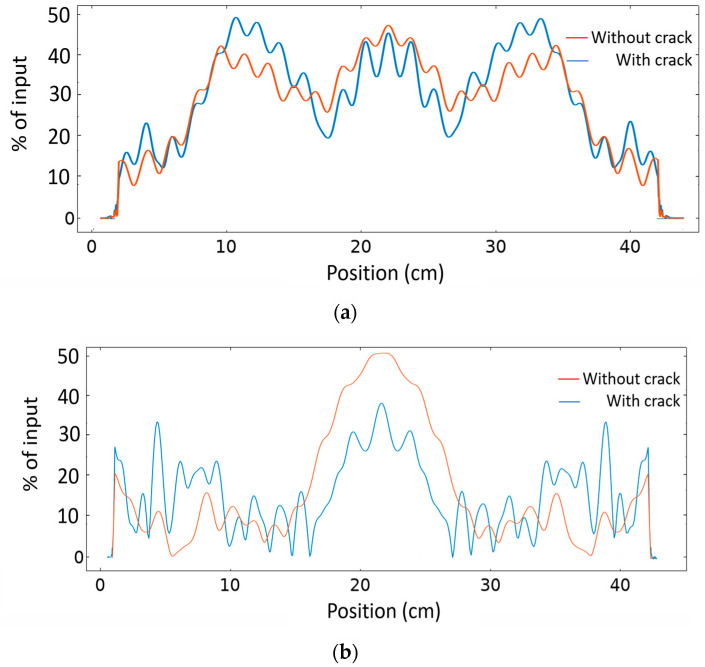
abs(u) with horizontal axis representing the position on a vertical line on the metallic sample at a distance 30 cm to the right of the crack, in case of (**a**) a sample without/with embedded crack when no PC lens is used (**b**) a sample without/with embedded crack when a PC lens is used.

Figure 12a show the surface plot of magnitude of the displacement field, abs(u), in the case of a normal diverging beam with no crystal, for a sample with an embedded elliptical crack. Multiple reflections and scattering at a wide angle can be seen. Figure 12b shows the surface plot of abs(u) when the PC is integrated between the ultrasound source and the sample under study. The collimated beam at 193 kHz is shows a reduced intensity and distorted propagation due to the presence of the crack in comparison with the case where no crack was present when we used the PC (Figure 8). Figure 13a shows the abs(u) intensity pattern on a vertical line (orange dashed line in Figure 11) at 30 cm spacing to the right of the elliptical crack where no PC lens is used at frequency 193 kHz in the case of healthy as well as in the case of the presence of a crack respectively. It can be seen there is a slight change in the intensity at the vertical position where the projection of the crack is located (5% reduction in the intensity at the position of crack projection (position = 20 cm)). However, when the PC lens was used and after monitoring the same vertical line’s intensity pattern with and without crack (Figure 13b), we can see a larger reduction in the intensity at the crack’s projection (29% reduction of intensity at vertical position = 20 cm). This is a significant improvement in crack detectability by the use of a PC lens in comparison with traditional techniques. In addition, the use of the PC helped avoid the boundary reflections coming from the boundaries of the target object and interfering with the scattered signal from the crack. Thus, we visualized quantitatively and qualitatively the significant improvement for NDT applications in solids when using a clean ultrasound wave propagation inside the material due to the use of an ultrasound PC lens.

We emphasize that, in the case where a PC is used, a scan of the ultrasound source must be applied to detect the defect location. This will be actually useful in case we want to detect multiple cracks adjacent to each other, each crack will be detected at a different scan point of the source when the PC is used, however, when a divergent source with no PC is used, the two defects will be recognized as one thing depending on the resolution of source/detector set, the divergence of the waves, and boundary reflections interference.

## 6. Conclusions

We have numerically studied the performance of a 2D phononic crystal device embedded in a metallic host for ultrasonic beam propagation control in solid materials, The PC is designed to provide both focalization and collimation of incident beams at different frequencies in their propagation behind the periodic structure. Our study considered 2D surface plots of the propagating waves across and behind the PC, in a metallic object and analyzed the frequency spectrum across chosen points and lines in order to understand the behavior of wave propagation in the near and far fields, at various angles. The frequency spectrum matches with the eigen frequency analysis using dispersion curves and equisurface contour maps of the allowed and forbidden frequencies. We achieve spatial focalization and self-collimation of ultrasound beams at different frequencies when they propagate behind the 2D phononic crystal. We show that these effects are preserved when the PC is detached from both the transducer and the solid media behind and we introduce coupling gel layers for the connection to the source and the potential media under study. Later, an integration over the typical frequency bandwidth provided by a transducer has been considered and the results proved that the focusing and collimation effects are also preserved. Finally, we have included here a study of a possible application for NDT industrial application and showed that the PC lens can provide improved results for detecting embedded cracks in the metal objects by reducing boundary reflections and providing a cleaner signal directed at the defect which enhances its detectability. NDT is a very large concept including multiple applications where the characterization of ultrasound waves in solids is needed. The localization of an embedded defect and the reconstruction of its shape based on acoustic image reconstruction are only a few examples. We highlighted one example that shows the utility and possible implementation of the proposed PC device for the improvement of the ultrasound propagation and NDT detection in a metallic target. The use of a PC device as a focusing/collimation lens for ultrasonic wave propagation in solids, similar to those presented here and with proper selection of crystal parameters and a matching coupling acoustic impedance, may be useful for different applications where a focused, nondiffractive, or spatially localized propagation inside the target is needed. Even though a further study of other possible applications is out of the scope of this paper, we are confident that this device opens the door to other technological applications.
